# Estimating the mode of inheritance in genetic association studies of qualitative traits based on the degree of dominance index

**DOI:** 10.1186/1471-2288-11-171

**Published:** 2011-12-21

**Authors:** Elias Zintzaras, Mauro Santos

**Affiliations:** 1 Department of Biomathematics, University of Thessaly School of Medicine, 2 Panepistimiou Str, Biopolis, Larissa 41110, Greece; 2 The Institute for Clinical Research and Health Policy Studies, Tufts Medical Center, Tufts University School of Medicine, 800 Washington Str, Boston, MA 02111, USA; 3 Departament de Genètica i de Microbiologia, Grup de Biologia Evolutiva, Universitat Autònoma de Barcelona, 08193 Bellaterra, Barcelona, Spain

## Abstract

**Background:**

The biological justification for the choice of the genetic mode in genetic association studies (GAS) is seldom available. Then, the mode of inheritance is approximated by investigating a number of non-orthogonal genetic contrasts making the interpretation of results difficult.

**Methods:**

We propose to define the mode of inheritance by the significance of the deviance of the co-dominant contrast and the degree of dominance (*h*), which is a function of two orthogonal contrasts (the co-dominant and additive). Non-dominance exists when the co-dominant contrast is non-significant and, hence, the risk effect of heterozygotes lies in the middle of the risk of the two homozygotes. Otherwise, dominance (including over- and under-dominance) is present and the direction of dominance depends on the value of *h*.

**Results:**

Simulations show that *h *may capture the real mode of inheritance and it is affected by deviations from Hardy-Weinberg equilibrium (HWE). In addition, power for detecting significance of *h *when the study conforms to HWE rule increases with the degree of dominance and to some extent is related to the mutant allele frequency.

**Conclusion:**

The introduction of the degree of dominance provides useful insights into the mode of inheritance in GAS.

## Background

Genetic association studies (GAS), candidate-gene and genome-wide association studies assess the association between a disease and genetic variants (gene polymorphisms) in a population [1]. For a bi-allelic candidate gene with alleles wild-type *wt *and mutant-type *mt *in a case-control study, where *mt *is thought to be associated with a disease, the association is usually assessed using a chi-squared test for the 3 × 2 contingency table with genotype entries *n*_11_(*wtwt*), *n*_21_(*wtmt*) and *n*_31_(*mtmt*) for the control subjects, and *n*_12_(*wtwt*), *n*_22_(*wtmt*) and *n*_32_(*mtmt*) for the diseased subjects [2]. However, besides evaluating the overall statistical significance, the clinical relevance of a genetic association depends on the magnitude of risk conferred to the carriers of allele *mt *[3]. Thus, in view of a significant association, the following contrasts of genotypes are defined by merging information of the genotype distribution [4], and estimated with the odds ratio (OR) and its 95% confidence interval (CI): (i) the additive contrast, which is defined as the comparison between the extreme genotypes -*mtmt *vs. *wtwt*- (in this contrast the heterozygotes are ignored. Note that this contrast does not correspond to the conventionally examined "additive model" which is tested using the Armitage's test for trend [5]); (ii) the recessive contrast, which compares genotype *mtmt *with the merged genotypes *wtwt *+*wtmt*; (iii) the dominant contrast, which merges genotypes *mtmt *+ *wtmt *and compares them with genotype *wtwt*; and (iv) the co-dominant contrast, which compares genotype *wtmt *against the merged genotypes *mtmt *+ *wtwt *[1].

The biological justification for the choice of the genetic contrasts (which may not necessarily present the genetic model of inheritance) to be tested is, however, seldom available and lack of *a priori *assumption for the specific genetic model is customary practice [6,7]. This actually translates to investigating all four models above in GAS, and the interpretation of results may be confusing; that is, it might be the case that more than one contrast or even all of them are statistically significant [1]. The obvious reason for this is because in a case-control 3 × 2 contingency table there are only 2 degrees of freedom (i.e., there are two independent contrasts at most) and, therefore, the previous contrasts are not statistically independent. Furthermore, when a large number of comparisons are made following a significant genotype effect, some of the contrasts might be significant due to a type I error.

In this paper, we propose an index (*h*) that measures the degree of dominance and allows inferring the mode of inheritance in GAS in a continuous scale. Then, we numerically analyze how the *h*-index performs by using of a population genetics model where the real mode of inheritance can be defined *a priori*, and provide estimates of power. We also investigate how deviations from Hardy-Weinberg equilibrium (HWE) can affect inferences of the mode of inheritance using the *h*-index. Finally, we illustrate the method with an empirical study of published GAS.

## Methods

### Contrast definition and model analysis

Consider a GAS of a bi-allelic polymorphism which evaluates the risk associated with allele *mt*. The genotype frequencies are given in a 3 × 2 contingency table with counts

(1) $$\begin{array}{ccc} \text{Genotype} & \text{Controls} & \text{Cases}\\ wt/)wt & n_{11} & n_{12}\\ wt/)mt & n_{21} & n_{22}\\ mt/)mt & n_{31} & n_{32} \end{array}$$

To analyze this case-control study, the following logit model can be fitted to the dataset

(2) $$\log\left(\pi \;/)\;1-\pi \right)\;=\;\beta_{0}\;+\;\beta_{1}\times g\;,$$

where *π *is the probability of being diseased, and *g* is the genotype effect with 3 levels. Then, the deviance due to the genotype effect (*D_g_*) in the model determines significance of *g*, and the significance test is based on the χ^2^-distributionwith 2 degrees of freedom (*df_g_*) [8].

Let us now define the additive and the co-dominant contrasts [1]. The additive model(*L_a_*) presents an individual contrast (comparison) between the two extreme homozygotes (with a single degree of freedom: $df_{L_{a}}=1$)

(3) $$L_{a}\;=\;\sum_{i}c_{i}g_{i}\text{ ;}\;c=\left[\begin{array}{ccc} 1 & 0 & -1 \end{array}\right]\text{ ; }\sum_{i}c_{i}=0\text{ ,}$$

and *g_i _*is the effect due to the *i*th genotype (*i *= 1, 2, 3) [9]. The magnitude of the association corresponding to this contrast is estimated by the natural logarithm(ln) of the odds ratio *θ_a _*= (*n*_32 _× *n*_11_)/(*n*_12 _× *n*_31_) (i.e., we refer to an additive contrast on the ln-scale). The estimator ln(*θ_a_*) is approximately normally-distributed with asymptotic variance $\text{Var}\left[\text{ln}\left(\theta_{a}\right)\right]=\left(\frac{1}{n_{32}}+\frac{1}{n_{11}}+\frac{1}{n_{12}}+\frac{1}{n_{31}}\right)$[10]. Statistical significance of the additive contrast can be tested using a *z*-test $z\;=\;\text{ln}\left(\theta_{a}\right)/)\sqrt{\text{Var}\left[\text{ln}\left(\theta_{a}\right)\right]}$.

The co-dominant model (L*_∞_*) is the individual contrast between the heterozygotes and the average of the two homozygotes (with $df_{L_{co}}=1$)

(4) $$\begin{array}{ccc} L_{co} & =\;\sum_{i}c_{i}g_{i},\; & \\ c & =\left[\begin{array}{ccc} -0.5 & 1 & -0.5 \end{array}\right]\text{ ; }\sum_{i}c_{i}=0\;\text{.} \end{array}$$

The magnitude of the association for this contrast is estimated as the natural logarithm of the odds ratio $\theta_{co}=\frac{n_{22}\times \left(n_{11}+n_{31}\right)}{n_{21}\times \left(n_{12}+n_{32}\right)}$, with asymptotic variance $\text{Var}\left[\text{ln}\left(\theta_{co}\right)\right]=\left(\frac{1}{n_{22}}+\frac{1}{n_{11}+n_{31}}+\frac{1}{n_{21}}+\frac{1}{n_{12}+n_{32}}\right)$. Statistical significance can also be tested using a *z*-test as above. The additive and co-dominant contrasts are clearly orthogonal because the dot product of the *c_i _*coefficients defining each contrast is zero (i.e., [1 0 -1]·[-0.5 1 -0.5] = 0) [9].

These statistically orthogonal contrasts can be interpreted separately since the deviance of the genotype effect can be split into two independent deviances: one due to the additive contrast $\left(D_{L_{a}}\right)$ and the other due to the co-dominant contrast $\left(D_{L_{co}}\right)$[8,9]. In other words, $D_{g}=D_{L_{a}}+D_{L_{co}}$, and $df_{g}=df_{L_{a}}+df_{L_{co}}$. Now the logit model (2) is equivalent to the model

(5) $$\log\left(\pi \;/)\;1-\pi \right)=\beta_{0}\;+\;\beta_{1}\times L_{a}+\;\beta_{1}\times L_{co}.$$

Testing the significance of the genotype effect is thus equivalent to simultaneously testing the significance of the additive and co-dominant contrasts based on the respective deviances or z-tests.

### Modelling the disease inheritance

**Figure 1:**
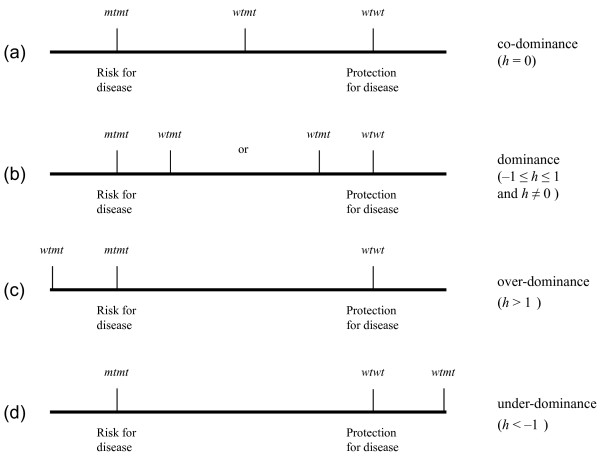
**Degree of dominance *h *is a function of the deviation in risk of disease of the heterozygote *wtmt *from the co-dominant model, in which the heterozygote is expected to lay exactly in the middle of the two homozygotes (a)**. Dominance deviation can be positive or negative (b), with the heterozygote closer to the risk-exposed homozygote *mtmt *(0 <*h *≤ 1), or to the risk-protected homozygote *wtwt *(-1 ≤ *h *< 0). Over-dominance (c) arises when the risk of disease of the heterozygote is higher than that of the risk-exposed homozygote (*h *> 1), whereas under-dominance (d) arises when the risk of disease of the heterozygote is lower than that of the risk-protected homozygote (*h *< -1).

We define here for a GAS showing a significant statistical association (i.e., showing significance for at least one of the two orthogonal contrasts) the model of disease inheritance according to the degree of dominance *h *of the mutant allele *mt*. In the extreme case where there is non-dominance (i.e., co-dominance or perfect additivity), the heterozygote *wtmt *"lies" exactly in the middle of the two homozygotes, with *mtmt *having the maximum susceptibility of being diseased and *wtwt* having the least (Figure 1). The deviance for the co-dominant contrast $\left(D_{L_{co}}\right)$ is then non-significant, whereas the deviance $\left(D_{L_{a}}\right)$ for the additive contrast is expected to be highly significant since we consider GAS with significant association. On the other hand, significance of $D_{L_{co}}$ indicates dominance (i.e., the heterozygote *wtmt *lies towards *mtmt *or *wtmt*) irrespectively of the significant level of $D_{L_{a}}$.

**Figure 2:**
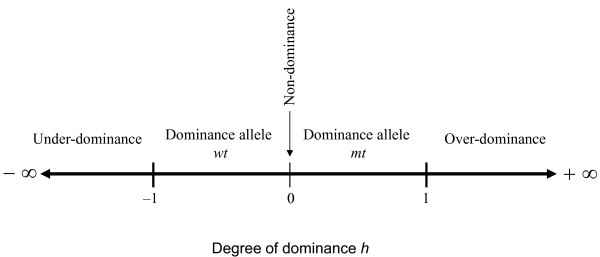
**Snapshot of the degree of dominance according to the sign of *h *in order to facilitate the interpretation of results**.

We can use now the odds ratios of the co-dominant and additive contrasts to define the magnitude and the direction of the degree of dominance. Deviation from perfect additivity can result in dominance (the heterozygote deviates from the middle of the two homozygotes), over-dominance (the heterozygote has a greater risk of disease than the homozygote *mtmt*), or under-dominance (the heterozygote has least risk for the disease than the homozygote *wtwt*) (Figure 1). Therefore, the degree of dominance could be derived from the ratio of the logarithms of the two previous odds ratios as

(6) $$h=\frac{\text{ln}\left(\theta_{co}\right)}{|\text{ln}\left(\theta_{a}\right)|}\text{ ,}$$

where "| |" denotes absolute value. In expression (6) the sign of ln(*θ_co_*) determines the direction of dominance, and the value of ln(*θ_co_*) relative to the absolute value of ln(*θ_a_*) the magnitude of dominance deviation *h*, which can take any value from negative infinity to positive infinity (Figure 2).

Once significance in dominance is detected and *h *is obtained, the degree of dominance is inferred as follows (assuming, of course, that homozygote *mtmt *has higher disease risk than homozygote *wtwt*) (Figures 1 and 2). If -1 ≤ *h *< 0 there is dominance of the wild-type allele *wt*, and the heterozygote *wtwt *is expected to have a risk of being diseased somewhere in between the middle of the two homozygotes and towards to the homozygote *wtwt*. If 0 <*h *≤ 1 there is dominance of the mutant allele *mt *and the heterozygote *wtmt *is expected to have a risk of being diseased somewhere in between the middle of the two homozygotes and towards to the homozygote *mtmt*. When *h *> 1 there is over-dominance and the heterozygote has a higher risk of being diseased than the homozygote *mtmt*, whereas if *h *< -1 there is under-dominance and the heterozygous has least chance of being diseased than the homozygote *wtwt*. However, over- and under- dominance is a rare phenomenon in GAS [6].

The statistical test to asses the significance of over- or under-dominance -that is, to test the null hypothesis H_0_: h = 1 *vs*. H_a_: *h *> 1 (over-dominance), or *vs*. H_a_: *h *< -1 (under-dominance) is equivalent to test:

i)$H_{0}\text{: }k=\text{ln}\left(\frac{\theta_{co}}{\theta_{\alpha }}\right)\text{ = }0$ vs. $H_{1}\text{: }k=\text{ln}\left(\frac{\theta_{co}}{\theta_{\alpha }}\right)>0$ (over-dominance) or $H_{1}\text{: }k=\text{ln}\left(\frac{\theta_{co}}{\theta_{\alpha }}\right)<0$ (under-dominance) when ln(*θ_α_*) > 0,

ii) *H*_0_: *k *= ln(*θ_co _*× *θ_α_*) = 0 vs. *H*_1_: *k *= ln(*θ_co _*× *θ_α_*) > 0 (over-dominance) or *H*_1_: *k *= ln(*θ_co _*× *θ_α_*) < 0 (under-dominance) when ln(*θ_α_*) < 0.

This can be done using a one-sided *z*- test where the variance of *k *is approximately $\text{Var}\left(k\right)=\text{Var }\left[\text{ln}\left(\theta_{co}\right)\right]\text{ + Var }\left[\text{ln}\left(\theta_{\alpha }\right)\right]\times \left(1-\frac{2}{\pi }\right),$ where (1-2/*π*) is arising from the variance of a half-normal distribution [11]. When the test is non-significant we conclude that there is just dominance or that over-/under-dominance is not beyond chance."

To summarize, inferences regarding any degree of dominance *h *are obtained from the following order of hypothesis. The first hypothesis states that there is non-dominance, and is tested using the co-dominant contrast. If this hypothesis is true, the risk of disease for the heterozygote *wtmt *is in the middle of the two homozygotes. If the co-dominant contrast is significant, we then test for the direction of dominance by stating the second hypothesis; that is, 0 < |*h| *≤ 1. If this hypothesis is true, the heterozygote *wtmt *has a risk of disease closer to the homozygote *mtmt *or the homozygote *wtwt *according to the sign (+ or -, respectively) of *h*. Finally, if this hypothesis is false we assess whether or not there is significant over- or under-dominance by testing the hypothesis |*h*| > 1 using *k*.

## Results

We suggest the use of *h *as defined in (6) to infer the mode of inheritance in GAS, but an obvious question is: how does this index perform? Here, we check (i) the performance of *h *when the real genetic model is known in advance from a standard population genetics model, then we analyze (ii) power from computer simulations by randomly exploring the parameter space, 0.2 ≤ *h *≤ 1.0 (-0.2 ≥ *h *≥ -1.0) in case-control studies, and (iii) the impact that deviations from HWE in the control subjects have on the *h *index.

### Performance of the h index

We make use of a standard one-locus, two-allele model in population genetics that generalizes the role played by the real degree of dominance (*H*) on the evolution of a deleterious mutant allele (*mt*) in a population [12] to analyze the performance of the proposed index, *h*. The population genetics model goes as follows

(8) $$\begin{array}{cccc} \text{Genotype} & \text{Frequencies }t_{1} & \text{Fitness} & \text{Frequencies }t_{2}\\ wtwt & p_{1}^{2} & 1 & \frac{p_{1}^{2}}{\bar{w}}\\ wtmt & 2p_{1}q_{1} & 1-Hs & \frac{2p_{1}q_{1}\left(1-Hs\right)}{\bar{w}}\\ mtmt & q_{1}^{2} & 1-s & \frac{q_{1}^{2}\left(1-s\right)}{\bar{w}} \end{array}$$

where $\bar{w}=p_{1}^{2}+2p_{1}q_{1}\left(1-Hs\right)+q_{1}^{2}\left(1-s\right)$, and *s *is the coefficient of selection (*s *> 0, i.e. *s *is the decrease in fitness of homozygous carriers for the mutant allele relative to the wild-type genotype). In words, we assume that there is an initial cohort of individuals in HWE and that in the time interval *t*_1_→*t*_2 _there is a genotype-dependent per capita probability of survival (relative to the wild-type homozygote *wtwt*) given by the values in the column labeled "fitness", with *s *> 0 and 0 ≤ *H *≤ 1; that is, we know in advance that allele *mt *is associated with an increased probability of death as time goes on. The parameter *H *captures the degree of dominance of the mutant allele, which is fully recessive when *H *= 0, co-dominant when *H *= 1/2, and fully dominant when *H *= 1 [13]. To convert this simple model into a hypothetical case-control study we proceeded as follows.

Assume 10,000 individuals at *t*_1 _with allele frequencies in the range 0.05 ≤ *q*_1 _≤ 0.5, and obtain the resulting genotype frequencies at time *t*_2 _in (8) for the whole range of the parameter space 0 ≤ *H *≤ 1 (changes in the parameter *s *do not affect the qualitative results). Because the "disease trait" we are studying is the genotype-dependent probability of death in the time interval *t*_1_→*t*_2_, the appropriate genotype distribution of cases to be compared with the controls (i.e., the initial cohort) is simply that arising from the individuals that have died. To avoid having entries with zero in the case-control study, we assumed a constant genotype-independent probability of death equal to 5% (this does not obviously affect the qualitative results). This procedure allows us to define parametric controls and cases for any type of inheritance mode (*H*) and selection coefficient (*s*). In order to simulate a "sampling" case-control study, *n *= 400/400 cases/controls subjects are sampled randomly form the parametric cohort based on the space defined by the cumulative genotype frequencies [e.g. if a control subject is randomly sampled in the space from *n*_1_/(*n*_1_+*n*_2_+*n*_3_) to (*n*_1_+*n*_2_)/(*n*_1_+*n*_2_+*n*_3_), then the subject is assigned as heterozygous].

**Figure 3:**
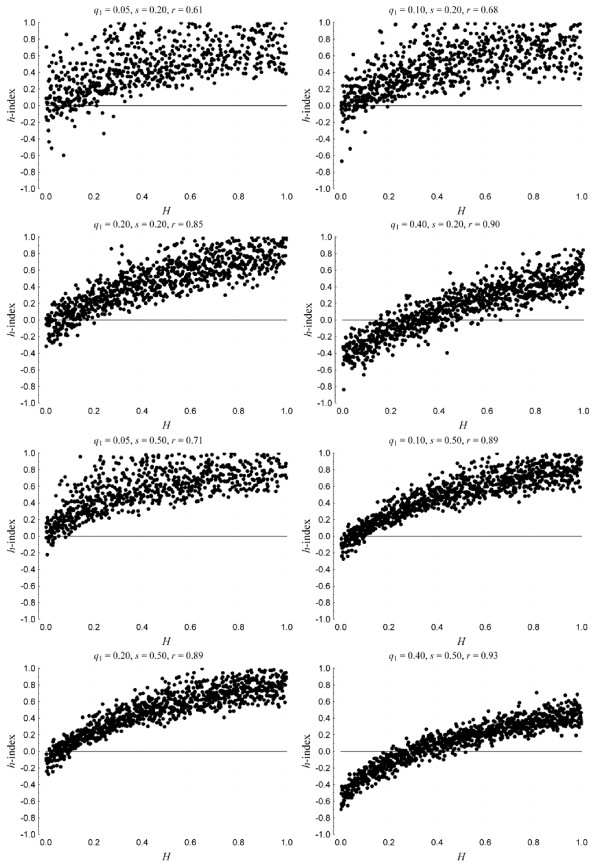
**Performance of the *h*-index against the true underlying mode of inheritance given by parameter *H *for allele frequencies in the range (0.05 ≤ *q*_1 _≤ 0.4 and selection coefficient *s *= 0.2 and 0.50, when 400/400 controls/cases are sampled from the parametric cohort**. Each plot also gives the Spearmann correlation between *H *and *h*-index.

Figure 3 plots index *h *against parameter *H *assuming *s *= 0.2 and 0.5. For very low allele frequencies (0.05 ≤ *q*_1 _≤ 0.40) there is little chance of detecting recessiveness of allele *mt *(i.e., *H *< 0.5) because, in general, *h *≥ -0.2; whereas for relatively high allele frequencies (*q*_1 _= 0.40), *h*-index can estimate more efficiently *H*. In any case, *h*-index tends to perform better when the selection coefficient increases (i.e. the risk of disease is higher in homozygous mutants).

### Power

To estimate power simulations were performed by randomly exploring the parameter space 0.1 ≤ *h *≤ 1.0 (0.1 ≥ *h *≥ -1.0) in case-control studies. Genotype frequencies *n*_*i*2_(*i *= 1, 2, 3) for the cases in (1) were randomly generated with the restriction $\underset{i}{\sum }n_{i2}=400$ and resulting frequencies for the disease-associated allele *mt *bounded by 0.09 ≤ *q*_cases _≤ 0.11, 0.09 ≤ *q*_cases _≤ 0.21, or 0.39 ≤ *q*_cases _≤ 0.41. Genotype frequencies in the controls were generated assuming HWE (*P *≥ 0.05) with the restriction $\underset{i}{\sum }n_{i1}=400$ and *q*_controls _≤ *q*_cases_. Power to detect dominance was assessed as [14]

(9) $$\text{Power}=\frac{\#\left(P<0.05\right)\text{ co - dominant contrast}}{\#\text{ simulations}}\text{.}$$

**Figure 4:**
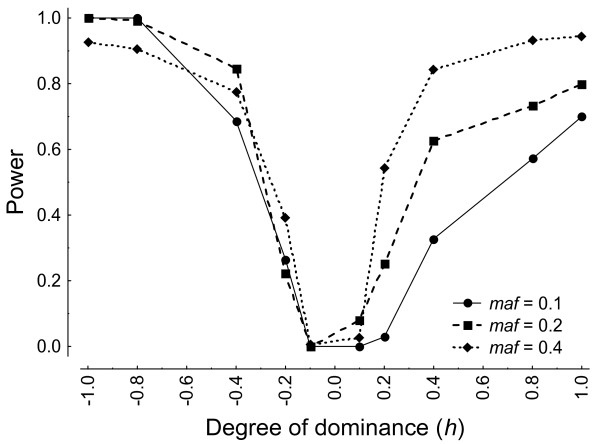
**Plot of power versus *h *assuming HWE in the controls *(P ≥ 0.05*) for different frequencies of the mutant allele (*maf*)**.

Figure 4 shows that power increases with increasing frequencies of allele *mt*, and also when *h *increases (decreases) as expected. There is, however, some asymmetry depending on allele *mt *being inferred to be dominant (*h *> 0; Figure 2) or recessive (*h *< 0). When *mt *is recessive power to detect moderate levels of dominance (*h *≤ -0.4) is substantial even at relative low allele frequencies, which might be explained due to the restrictions imposed to genotype frequencies.

### Impact of deviations from HWE on h index

We now analyze how estimates of the degree of dominance *h *in GAS are affected by deviations from Hardy-Weinberg equilibrium (HWE) in the control subjects [15]. We first present a general analytical treatment on the topic, and then illustrate the analysis with simulations.

Following Weir [16], genotype frequencies in the controls can be modeled from (1) as *n*_11_= *n*_•1_× (*p*^2^+ *D*), *n*_21_= *n*_•1_× (2*pq*- 2*D*) and *n*_31_= *n*_•1_× (*q*^2^+ *D*); where *p *(q = 1- *p*) is the frequency of allele *wt*(*mt*), *n*_•1_= *n*_11_+*n*_21_+*n*_31 _is the total number of control subjects in the study, and *D *$\left(D=\frac{n_{11}}{n_{∙1}}-p^{2}\right)$ is the Hardy-Weinberg disequilibrium parameter which is expected to be zero when a population has Hardy-Weinberg proportions (i.e., testing for HWE is equivalent to a test of the hypothesis H_0_: *D *= 0). Bounds on *D *are [17]

(10) $$\max\left[-p^{2},-q^{2}\right]\;\leq D\;\leq pq\text{.}$$

Additive and dominant contrasts can now be generally written as

(11) $$\begin{array}{ccc} \theta_{a} & =\left(\frac{n_{32}}{n_{12}}\right)\times \left(\frac{p^{2}+D}{q^{2}+D}\right) & \\ \theta_{co} & =\left(\frac{n_{22}}{n_{12}+n_{32}}\right)\times \left(\frac{p^{2}+q^{2}+2D}{2pq-2D}\right). \end{array}$$

From (10) it is straightforward to estimate the impact deviations from HWE will have in the two contrasts (reasonably assuming *p *≥ *q*)

(12) $$\begin{array}{c} \frac{n_{32}}{n_{12}}\times \frac{p}{q}\;\leq \theta_{a}\left(\text{HWE}\right)\;\leq \infty \\ \infty \geq \theta_{co}\left(\text{HWE}\right)\;\geq \left(\frac{n_{22}}{n_{12}+n_{32}}\right)\text{,} \end{array}$$

where the first terms in the inequalities are for a positive value of *D *(*D = pq*, which obviously implies that there is an excess of homozygotes), and the last terms are for negative values (*D = -q*^2^, with an excess of heterozygotes). From (12) it is now clear that population stratification (i.e., when a sample is composed of sub-samples that differ in allele frequencies, thus generating and excess of homozygotes due to the well-known Wahlund's principle in population genetics [12]) will generally upward bias the absolute value of *h*, where the opposite is true when there is an excess of heterozygotes in the controls.

**Figure 5:**
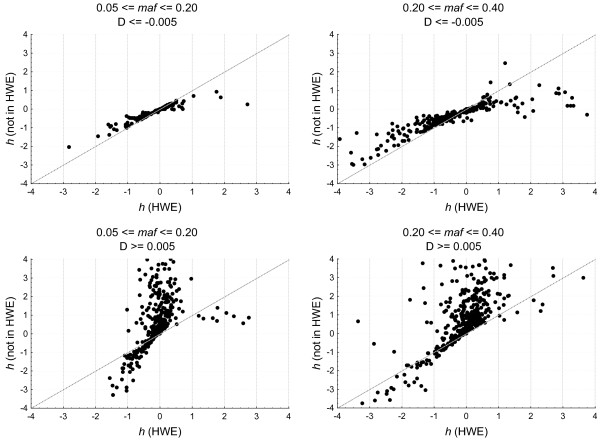
**Plot of the effect deviation from HWE have on *h *when there is excess of heterozygotes (*D *< 0) or an excess of homozygotes (*D *> 0) in the controls**. Only values in the range (-4, 4) are plotted for different frequencies of the mutant allele (*maf*).

Simulations were performed by randomly generating subject cases with genotype frequencies *n*_*i*2 _(*i = *1,2,3) under the restriction $\underset{i}{\sum }n_{i2}=400$ and 0.05 ≤ *q*_cases _≤ 0.40 for allele *mt*. Genotype frequencies in the controls were modeled following Weir [16] as indicated above, with the restriction *q*_controls _≤ *q*_cases _and *D *≥ 0.005 (*D *≤ -0.005). Note that when modeled in this way we are assuming parametric genotype frequencies in the controls; that is, we assume perfect HWE in the controls with allele frequencies equal to the simulated values. Figure 5 illustrates the bias incurred when estimating *h*, and also points to an asymmetry in the sense that within the range -1 ≤ *h *≤ 1 studies where *D *< 0 could perhaps be included without the bias being too serious (note, however, that in some cases *h *changes sign), but when *D *> 0 occurs we may not capture the true direction of dominance.

We now illustrate the proposed methodology by applying it to three working examples. Thereafter, an empirical study using a database of 831 GAS was carried out.

### GAS with non-dominance

A GAS investigating the association between the *ACE D/I *polymorphism and coronary artery disease (CAD) produced the following genotype distributions [18]:

$$\begin{array}{ccc} \text{Genotype} & \text{Controls} & \text{Cases}\\ II & \text{64 (30}\text{.6\% )} & 34\;\text{(16}\text{.6\% )}\\ ID & 94\;\text{(45}\text{.0\% )} & 89\;\text{(43}\text{.4\% )}\\ DD & \;51\;\text{(24}\text{.4\% )} & 82\;\text{(40\% )} \end{array}$$

After fitting the logit model (2) the change in deviance due to the genotype effect was *D_g _*= 16.72 with *df_g _*= 2 (*P *< 0.01); thus, there is significant association between *ACE D/I *polymorphism and CAD. Now, we expect at least one of the orthogonal contrasts to be significant. When the genotype effect was split into its two independent contrasts and the logit model was re-fitted following equation (5), the changes in deviance were $D_{L_{a}}=16.62$ with $df_{L_{a}}=1$ (*P *< 0.01), and $D_{L_{co}}=0.10$ with $df_{L_{co}}=1$ (*P *= 0.75), for the additive and co-dominant contrasts; respectively. $D_{L_{a}}+D_{L_{co}}=16.72$ as expected because the two degrees of freedom of the genotype effect were orthogonally decomposed into its two genetic components. Given that $D_{L_{co}}$ was not significant the data suggest that the mode of inheritance can be non-dominance. The interpretation would then be that the heterozygote *ID *has a risk of being diseased that lies in the middle of the risk-protected *II *and risk-exposed *DD *homozygous genotypes.

### GAS with dominance

A GAS investigating the association between the alleles ADH2*1 and ADH2*2 with alcoholism produced the following genotype distributions [19]:

$$\begin{array}{ccc} \text{Genotype} & \text{Controls} & \text{Cases}\\ {}*2/)*2 & 448\;\text{(82}\text{.2\% )} & 238\;\text{(70}\text{.0\% )}\\ {}*2/)*1 & 93\;\text{(17}\text{.1\% )} & 85\;\text{(25}\text{.0\% )}\\ {}*1/)*1 & 4\;\text{(0}\text{.7\% )} & 17\;\text{(5}\text{.0\% )} \end{array}$$

The logit model with the genotype effect was fitted and the deviances were significant for both contrasts (*P *< 0.01 for $D_{L_{co}}$ and $D_{L_{a}}$). Because the co-dominant model is significant, we proceed to inquiry about the degree of dominance, which here is *h *= ln(*θ_co_*)/|ln(*θ_α_*)| = 0.48/|2.08| = 0.23, suggesting dominance for the risk-associated allele *1. Therefore, we may conclude that the homozygous *1/*1 has a greater risk of being alcoholic than the homozygous *2/*2, and that the heterozygote *2/*1 has a risk of alcoholism closer to the *1/*1 homozygote than to the midpoint between the two homozygotes.

### GAS with under-dominance

A GAS investigating the association between the *BDNF G196A *polymorphism and schizophrenia produced the following genotype distributions [20]

$$\begin{array}{ccc} \text{Genotype} & \text{Controls} & \text{Cases}\\ GG & 208\;\text{(59}\text{.4\% )} & 229\;\text{(71}\text{.3\% )}\\ GA & \text{131 (37}\text{.4\% )} & \text{83 (25}\text{.9\% )}\\ AA & \text{11 (3}\text{.1\% )} & 9\;\text{(2}\text{.8\% )} \end{array}$$

In this last example, the co-dominant contrast was significant (*P *< 0.01) whereas the deviance for the additive contrast was not (*P *< 0.52). The degree of dominance is *h *= ln(*θ_co_*)/|ln(*θ_α_*)| = -0.54/|0.30| = -1.82, therefore there is indication for under-dominance since *h *< -1. The one-sided *z*-test for under-dominance (*h *< -1 or *k *< 0or) is statistically significant (*P *= 0.02). Thus, it seems that the heterozygote *GA *has the least risk of disease (or higher protection) than both homozygotes, which do not statistically differ between them.

### Empirical study

A database of 831 GAS archived in the Department of Biomathematics, University of Thessaly (http://biomath.med.uth.gr/), was utilized for the empirical study [15]. A GAS was considered eligible when (i) it examined bi-allelic polymorphisms; (ii) it provided the complete genotype distribution for diseased subjects and controls of individual studies included in the meta-analysis; (iii) controls were non-diseased; (iv) it was written in English; and (v) considered binary outcomes.

In 208 GAS, the genotype distribution showed a significant association after fitting the logit model (2) with the genotype effect. These studies involved 58 variants from 20 genes in association with 12 phenotypes. Table 1 summarizes the inferred degree of dominance (*h*) in the GAS, and groups studies according to the mode of inheritance (non-dominance, dominance, over-dominance, and under-dominance), and also according to the significant associations found for the contrasts that were of customary used in previous analyses.

**Table 1:** Results from 208 GAS studies that render a statistically significant association.

Degree of dominance	Total (*)	Number of GAS with significant contrasts			
		Co-dominant	Additive	Dominant	Recessive
Non-dominance (*h *= 0)	114	0	114	64	86
Dominance (*h*≠0)	94	94	22	59	29

Under-dominance (*h *< -1)	26(18)	26	1	10	9
Dominance (-1 ≤ *h *< 0) or (0 <*h *≤ 1)	41	41	21	34	16
Over-dominance (*h *> 1)	27(15)	27	0	15	4

The degree of dominance is presented. The results produced by applying the dominant and recessive contrasts are also shown.

* Studies with significant (*P *< 0.05) under- or over-dominance

The important point here is that in most GAS (114 out of 208; i.e., 55%) the proposed method defines a non-dominance inheritance, whereas the conventional contrasts applied to these studies would render a statistical significant dominant contrast (64 out of 114; i.e., 56%) or recessive contrast (86 out of 114; i.e., 75%). However, 19% (i.e., 18 out of 94) of the studies with dominance deviated from HWE with *D *> 0 and 20% with *D *< 0; and with non-dominance the values are 3% (i.e., 3 out of 114) with *D *> 0 and 9% with *D *< 0. The majority of studies with non-dominance (86%) or dominance (84%) were underpowered (power < 0.5); and only 6% and 5%, respectively, have power > 0.8.

**Figure 6:**
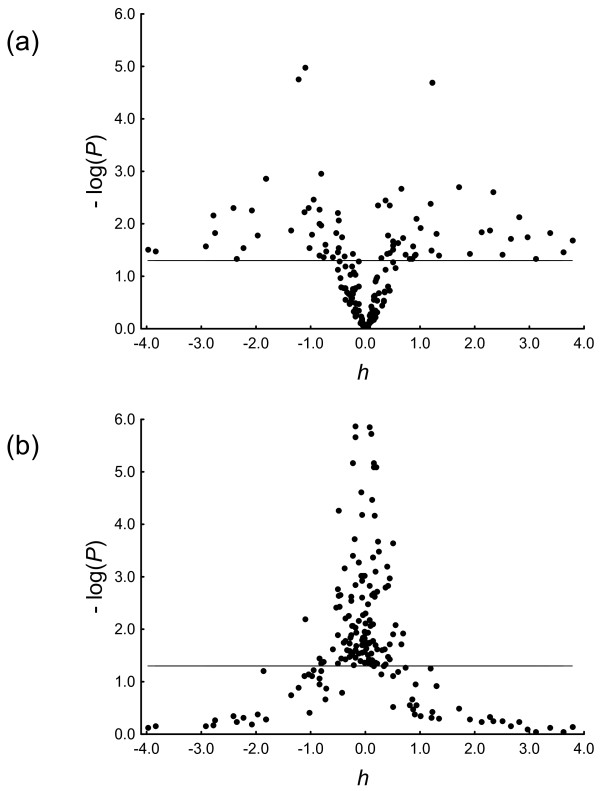
**Empirical behavior of the degree of dominance *h *from 208 GAS showing significant genotype effects**. The plots show -log[*P*] as a function of *h *for (a) the co-dominant and (b) the additive contrasts, and the horizontal line at -log[*P*] = 1.3 represents the critical value above which statistical significance (*P *< 0.05) is attained. There is a clear reverse behaviour in the plots and, as predicted from our numerical study, statistical significance of the additive model was found when under- or over-dominance were absent (i.e., |h| ≤ 1).

Also important is that in a significant proportion of studies (25%) our method detects under- (12%) or over-dominance (13%). Figure 6 plots -log[*P*] as a function of *h *for the co-dominant and the additive contrasts. Statistical significance of the additive model was (with a few exceptions) only found when under- or over-dominance were absent.

## Discussion

Herein, we have proposed to identify the mode of inheritance in a continuous scale using the degree of dominance *h*, which is based on the ratio of the odds ratio of the co-dominant contrast divided by the absolute value of the odds ratio of the additive contrast. Numerical results suggest that the *h *index captures the essence of what should be understood by genetic model or mode of inheritance. A meticulous analysis has been performed to check performance against an *a priori *model where we already know that a mutant allele is associated to a disease and also the degree of dominance of this allele. Simulations also show that the degree of dominance *h *is affected by deviations from HWE, although the bias is more serious when there is population stratification. In these cases the findings should be interpreted carefully, and adjustments for departures from HWE might be applied [1,10]. Furthermore, power for detecting significance for *h *when the study conforms HWE rule increases with the degree of dominance and to some extent is related to the mutant allele frequency.

The empirical study we carried out showed the degree of dominance may sufficiently indicate the mode of inheritance. Any degree of dominance exists when the co-dominant contrast is significant irrespectively to the additive contrast. The co-dominant and additive contrasts show a reverse pattern in *h *and, also important, in the range of over- or under-dominance the additive contrast is non-significant. In general, candidate-gene studies have a tendency to lack power for detecting dominance arising from weak genetic contributions of common variants; though, large genome-wide association studies have been undertaken recently and an effort to create consortia for data sharing is initiated [21,22]. An underpowered GAS will underestimate the significance of the orthogonal contrasts and, therefore, the value of *h*. Nevertheless, the power to detect the significance of the co-dominant contrast and/or the additive contrast is the same with the power to detect a significance association between the genotype distribution and the disease using a logistic regression with explanatory variable the genotype with three levels. The proposed index may be applied to both types of GAS (candidate-gene studies and GWAS) in the same way (of course the recording of the genotype distribution is a necessary condition). However, in testing the significance of the orthogonal contrasts for an individual variant of a GWAS a multiplicity adjustment should be considered.

In order to avoid the obstacle of the multiple genetic contrasts, Zintzaras [4] proposed the concept of generalized odds ratio (OR_G_) for analyzing and meta-analyzing GAS. OR_G _is a single statistic that summarizes the magnitude and significance of the association without considering the hash of possible contrasts, and provides a straightforward interpretation of the results in GAS. The OR_G _utilizes the complete genotype distribution and provides an estimate of the magnitude of the association, given that the mutational load and the phenotype (bi-allelic or multi-allelic) are treated as a graded exposure (case-control or disease progression). Specifically, OR_G _express the probability of a subject being diseased relative to probability of being free of disease, given that the diseased subject has a higher mutational load than the non-diseased. OR_G _with values greater than one suggests that disease is proportional to increased genetic exposure and inversely proportional for values less than one. Thus, the application of OR_G _may overcome the shortcomings of multiple model testing or erroneous model specification and provides an alternative and robust way for genetic association testing.

Regarding meta-analysis, model-free approaches have been proposed to estimate the genetic model [6,23]. However, we would like to stress that merging studies with potentially heterogeneous modes of inheritance should be avoided since we could entirely miss the true biological meaning underlying disease susceptibility. The application of our proposed method to identify the mode of inheritance warrants further investigation in this context. Although the methodology proposed here is straightforward, a Bayesian approach for implementing the method might be more desirable, especially when there is prior estimation of the magnitude and accuracy of the genetic risk effect and the degree of dominance [24,25].

## Conclusion

The introduction of the degree of dominance *h*, which is the ratio of the two orthogonal contrasts, may provide useful insights into the mode of disease inheritance.

## Competing interests

The authors declare that they have no competing interests.

## Authors' contributions

Both authors contributed equally to this paper and they read and approved the final manuscript.

## Pre-publication history

The pre-publication history for this paper can be accessed here:

http://www.biomedcentral.com/1471-2288/11/171/prepub
